# Remote consultations in mental health: collaborative evaluation applying learning health systems thinking

**DOI:** 10.1192/bjb.2024.102

**Published:** 2025-12

**Authors:** Lucy Goulding, Julie Williams, Alison White, Aileen Jackson, Zoë Lelliott, Stuart Adams, Kia-Chong Chua, Noushig Nahabedian, Juliana Onwumere, James Woollard, MOMENT group, Abdullah Aisha, Adams Stuart, Casetta Cecilia, Kia-Chong Chua, Cope Sarah, Curran Natasha, Davies Lydia, Demetriou Len, Finch Emily, Fonagy Peter, Gaughran Fiona, Melanie Getty McManus, Goulding Lucy, Graham Elizabeth, Grey Barbara, Jackson Aileen, Jordan Harriet, Lawrence Robert, Lelliott Zoe, Lennon Paul, Lloyd Alex, Lowry Sophie, Markham Sarah, Nahabedian Noushig, Onwumere Juliana, Pearson Nina, Jacqueline Phillips Owen, Samuels Lana, Sevdalis Nick, Walker Andrew, White Alison, Williams Julie, Wojewodka Gabriella, Woollard James, Appleton Rebecca, Badhan Monika, Barnett Phoebe, Zoë Haime, Jasmine Harju-Seppänen, Johnson Sonia, Brynmor Lloyd-Evans, Mo Jiping, Needle Justin, Papamichail Alexandra, Roxburgh Emily, Norha Vera San Juan, Schlief Merle, Spyridonidi Spyros, Luke Sheridan Rains, Simpson Alan, Steare Thomas, Tomaskova Magdalena, Lisa Wood, Nick Sevdalis, Fiona Gaughran

**Affiliations:** 1 King's College London, London, UK; 2 Health Innovation Network, London, UK; 3 South West London and St George's NHS Trust, London, UK; 4 South London and Maudsley NHS Foundation Trust, London, UK; 5 Oxleas NHS Foundation Trust, London, UK

**Keywords:** Remote consultations, evaluation, mental health services, learning healthcare systems, collaboration

## Abstract

**Aims and method:**

A collaborative evaluation of remote consultations in mental health services was undertaken by mental health service providers, experts by experience, academic institutions and a Health Innovation Network in south London, UK. ‘Learning healthcare systems’ thinking was applied. Workstream 1 reviewed international published evidence; workstream 2 synthesised findings from three health provider surveys of the perceptions and experiences of staff, patients and carers; and workstream 3 comprised an electronic survey on local projects.

**Results:**

Remote consultations can be acceptable to patients and staff. They improve access for some while restricting access for others, with digital exclusion being a key concern. Providing tailored choice is key.

**Clinical implications:**

The collaboration generated learning to inform choices by healthcare providers to embed or adapt remote delivery. A key output was freely downloadable survey questions for assessing the quantity and quality of appointments undertaken by phone or video or face to face.

The coronavirus SARS-CoV2, which causes the respiratory illness COVID-19, was first identified in late 2019. A pandemic was declared by the World Health Organization on 11 March 2020. To reduce infection, social distancing measures were introduced by countries around the world to limit the spread of the virus. On 23 March 2020, the first lockdown, ordering people to ‘stay at home’, was announced in the UK. In accordance, mental health services rapidly increased their adoption of remote consultations between staff and patients, utilising video conferencing platforms and telephone calls to minimise face-to-face contact and travel. Many services transitioned quickly from rarely or never offering remote consultations to this being common practice. This drastic change meant that, effectively, numerous natural experiments were taking place across the world and across all types of health service. This raised important questions and challenges. A key question was whether remote delivery was feasible and desirable to the target end user and for staff groups to implement and to what extent it was safe and effective (both as a crisis substitute for and when compared with face-to-face delivery). A challenge was the speed of implementation, dictated by a public health necessity, which precluded controlled evaluation design. Evaluation of remote mental (and other) health service delivery emerged as a key objective, in light of the scale and implications of service changes.

The published literature relevant to remote consultations in mental health services was synthesised and summarised as part of the findings presented in this paper.

## Method

### Learning healthcare systems approach

In this paper, we focus on the collaborative evaluative approach taken in mental health service provision across south London, UK. The region represents a catchment area of 12 London boroughs, serving approximately three and a half million people from diverse ethnic and socioeconomic backgrounds. South London hosts several major mental health service providers, academic institutions, and a Health Innovation Network (HIN), which is a National Health Service (NHS) body tasked with identifying, spreading and adopting evidence-based health and healthcare innovations. Specifically, in response to the requirement for rapid change, in spring 2020, representatives from the three NHS mental health trusts in south London, UK, members of the public with lived experience of using mental health services (experts by experience), academics and the local HIN collaborated on a project designed to bring together both local and international information to learn what was known and unknown about remote consultations in mental health, to share this information to help mental health services and patients to understand the evidence base, and to apply this to service planning delivery, as well as to identify knowledge gaps. This multi-institutional partnership was termed the MOMENT group (reMOte MENTal health).

In broad terms, the group applied ‘learning healthcare systems’ thinking to the project; this was defined by the Institute of Medicine (now the National Academy of Medicine) as an approach in which ‘science, informatics, incentives, and culture are aligned for continuous improvement and innovation, with best practices seamlessly embedded in the delivery process and new knowledge captured as an integral by-product of the delivery experience’.^1^ In short, the approach facilitates information sharing and rapid uptake of the newest evidence.^2^ Romanelli et al. describe learning healthcare systems as being ‘an ideal organizing principle to inform a unified and data-driven response to national public health emergencies like COVID-19’.^3^ In this paper, we describe the approach used by the group to create a community of learning during a time of crisis and present synthesised findings from three workstreams that were developed and delivered by the MOMENT group to evaluate the delivery of remote consultations in mental health services across organisations providing mental health services in south London, UK.

### Formation of the MOMENT group

The MOMENT group comprised a multidisciplinary team of project managers, experts-by-experience, clinicians, health service managers and researchers that convened in April 2020 to gather and share evidence on remote working. The original impetus for the formation of MOMENT came from senior managers, clinicians and academics in the region, discussing and reflecting on the early impacts of the pandemic, including the shift from face-to-face patient meetings to remote working, the speed of the shift and the new model of healthcare delivery. This aligned with a stated priority for NHS England at the time, both nationally and within the London region.

The south London context was considered a facilitator, in that the region afforded the key elements of a learning health systems approach, including: large and varied mental health service providers with clear needs; one of the largest global concentrations of mental health and implementation science research experts; the presence of regional innovation brokers (i.e. the HIN); a developed tradition of engaging experts by experience; and a trusting and supportive web of relationships between senior leaders across these organisations, which facilitated transparent information sharing and communications. The partner organisations are listed in Table 1. Members from each organisation met regularly (fortnightly to monthly as needed) between April 2020 and September 2021 to facilitate delivery of the project. Experts by experience from existing research groups helped in setting up the project, and four colleagues with lived experience formally joined the MOMENT group and attended meetings from September 2020 onwards.

**Table 1:** Membership of the MOMENT group^a^

Organisation name	Organisation type
Applied Informatics Theme, National Institute for Health Research Applied Research Collaboration South London	Academic partner
King's Improvement Science at King's College London	Academic partner and a programme of King's Health Partners academic health science centre
Centre for Implementation Science at King's College London	Academic partner
Health Innovation Network South London	Health innovation network
UCLPartners	Academic health science centre and health innovation network
Oxleas NHS Foundation Trust	NHS mental health trust
South London and Maudsley NHS Foundation Trust	NHS mental health trust
South West London and St George's NHS Trust	NHS mental health trust

a. Four public members with lived experience of using mental health services were an integral part of the MOMENT group.

### Aim and objectives

The overall aim of the MOMENT group was to better understand the impact of remotely delivering consultations in mental health services, and what has worked or been less successful and for whom, to inform choices by healthcare providers to embed or adapt remote delivery going forward to ensure the greatest benefits for patients, carers and staff.

The specific objectives were to:

1. systematically appraise the international literature on remote consultations in mental health services to demonstrate what is known about effectiveness, cost-effectiveness, implementation, and experiences of patients, carers and staff;
2. better understand access to and potential exclusion from remotely delivered services and the perspectives and experiences of remote consultations of those using or working in mental health services in south London during the first phase of the COVID-19 pandemic;
3. gather information about projects within local mental health services that were examining remote working to understand what data were being collected and to facilitate sharing of information across services and organisations;
4. triangulate findings from this international and local body of work to identify commonalities and areas of difference, highlight gaps in the evidence base around remote consultations in mental health services which may need to be addressed in future research, and support mental health service providers, patients and carers to learn what is known about remote consultations.

### Workstreams

Three workstreams were created to address objectives 1–3.

#### Workstream 1

Workstream 1 synthesised the international published evidence base on remote consultations within mental health services. Two systematic literature reviews were conducted: a rapid umbrella review (also known as a review of reviews) of systematic reviews published before the COVID-19 pandemic (search dates 1 January 2010 to 26 August 2020); and a systematic review of literature published during the COVID-19 pandemic (search dates 1 January 2020 to 9 December 2020). The methods and findings of the two systematic reviews have been published in full elsewhere.^4,5^

#### Workstream 2

Workstream 2 thematically analysed the findings presented in reports of healthcare provider organisation-wide surveys on the perceptions and experiences of staff, patients and carers that were designed, disseminated and analysed by the three collaborating mental health trusts (data collected between April and August 2020). This was a secondary analysis and synthesis of anonymous survey findings.

#### Workstream 3

Workstream 3 comprised a tailor-made electronic survey to collect information about planned evaluation, research or quality-improvement projects studying any aspect of remote working (both patient-facing and interprofessional) within mental health services during the first wave of the pandemic (data collected between July and November 2020). The survey was initially carried out in south London, and then extended to north-east and north-central London, UK. The focus of the survey was to collate information about project aims and methods.

### Synthesis of findings across workstreams

Objective 4 was met via a thematic analysis^6,7^ and synthesis of the findings across the three workstreams. This involved the following steps: researchers (L.G., J.W., N.S. and F.G.) read through the data and findings from workstreams 1–3 to re-familiarise themselves with the subject matter and develop a deep understanding of its content and context. The researchers wrote summary memos and/or notes, which were discussed and compared among team members. Preliminary headings (codes and/or labels) were noted to describe the content of the findings. Interpretations of these codes were discussed among the research team and then with the wider MOMENT group. Patterns in the headings were then categorised into overarching themes using an inductive approach. Both frequently observed concepts and those that were reported less often within the workstream findings but were salient to understanding diverse findings and perspectives were examined. Comparisons were made to examine similarities and differences in themes across the three workstreams. The emerging themes were reviewed by the MOMENT group and subsequently refined over two iterations to create a report of the synthesised findings across the workstreams.

### Use of models, theories or frameworks

The MOMENT group took a pragmatic view of the use of relevant theories to support evolving thinking and how to manage the large and varied data-sets that the project generated in a rapid manner. Theories were selected based on specific needs of different workstreams of the project, with the aim of helping to frame questions that the group had, or data analyses. As a general framework for this work, the MOMENT group drew upon learning healthcare systems thinking to rapidly gather and share information across the mental healthcare system in south London. Further, the academic partners within the collaboration drew upon well-established implementation science methods and frameworks within some of the workstreams. Specifically, the systematic review of literature published during the acute phase of the COVID-19 pandemic used the Consolidated Framework for Implementation Research^8^ and a taxonomy of implementation outcomes^9^ to structure data extraction. One of the organisation-wide surveys of staff and patients used validated brief implementation scales to assess perceived acceptability, appropriateness and feasibility of remote care delivery.^10^ Within workstream 3, we sought to capture data on the use of theories, frameworks and models being used to inform project design or analysis.

### Dissemination of learning across the system

Dissemination of project findings included presentations during the regular MOMENT group meetings, delivery of three workshops attended by wider audiences (1050 people registered, with many more watching recordings of the workshops later), development and sharing of infographics led by the expert-by-experience members of the team, and the creation of a project webpage to share learning across the system and beyond.^11^

### Ethics statement

The NHS trusts involved (Oxleas NHS Foundation Trust, South London and Maudsley NHS Foundation Trust, and South West London and St George's NHS Trust) each approved the project and associated data collection locally as a service evaluation exempt from NHS ethics approval.

## Results

### Summary of findings

#### Systematic evidence reviews (workstream 1)

The umbrella review of research literature published before the COVID-19 pandemic synthesised findings across 19 systematic reviews.^4^ Findings suggest that remotely delivered mental health services can be as efficacious and acceptable to staff and patients as face-to-face formats, at least in the short term. However, there was little evidence on large-scale implementation of remote working and effectiveness in ‘real-world’ (i.e. outside a research study) settings. Further, the findings of the umbrella review did not provide evidence relating to digital exclusion and how it might be overcome and were not able to provide conclusions on particular service contexts.

The rapid systematic review of COVID-19 specific literature synthesised 77 relevant papers published within the agreed timeframe.^5^ These studies were all conducted in the global north (USA, UK, Australia, Canada and Spain). Findings demonstrated that many of these countries had been able to rapidly shift to remotely delivered mental health services as a result of the pandemic. In general, studies suggested that remote appointments were reasonably well accepted, particularly where the alternative was no contact. A mixture of telephone and video-based calls were offered, with people expressing different preferences for these. Concerns about remotely delivered services were raised in relation to new patients, physical healthcare, and privacy and confidentiality. There was a distinct lack of information within the studies on whether active attempts were made to reach those at risk of digital exclusion; thus, the needs and experiences of those with poor or no access to remote consultations remain unknown. A small number of studies formally investigated implementation. Suggestions to improve implementation included staff training, champions for remote working, providing patients with access to technology and guidance on how to use it, and providing staff with guidance on identifying whether a remote offer is appropriate in different situations and/or with different individuals. Overall, the literature suggests that the delivery of remote consultations has been largely successful within the context of a pandemic, but longer-term evaluation is needed.

#### Surveys of patients, carers and staff (workstream 2)

The thematic analysis included results from two patient and carer surveys, one survey of consultants and specialty and associate specialty doctors, and one survey that collected responses jointly from patients, carers and staff (Table 2). The themes produced were considered according to whether the survey responses had been collected from patients or staff. Four overarching themes were derived. Reports of survey findings are published online.^11^

**Table 2:** Details of the organisation-wide surveys

	Oxleas	South London and Maudsley	South West London and St George's – patients	South West London and St George's – staff
Population surveyed	Patients in contact with adult mental health, adult community health, children and young people, older adults mental health and adult learning disability services	Staff, patients and carers across all trust directorates (all received the same survey)	Patients using the Attend Anywhere platform in all services	Consultants and specialist grade doctors
Aim of survey	To assess how Oxleas NHS patients and users of services feel about the change to remote consultations due to the pandemic	To find out about staff, patient and carer experiences of virtual appointments and/or meetings	To assess patient experience of using Attend Anywhere, including any potential financial and environmental impacts, to support the development of a formal business case for video consultation	To understand how staff had adapted to the changes in working practices due to the pandemic and the impact these had on productivity and well-being
Format(s) of survey	Online	Online	Survey in a weblink embedded in the Attend Anywhere platform	Online
Timeframe	15 March 2020−31 July 2020	29 April−23 June 2020	17 June−24 August 2020	11−24 June 2020
Method(s) of distribution	By SMS and email, or by phone for older adults or people with learning disabilities	For staff: weekly newsletter, trust intranet, leadership teams, trust broadcast. For patients and carers: patient and public involvement networks, patient and carer leads, patient and carer meetings, SLaM Recovery College	Weblink added to all Attend Anywhere calls	Not reported
Number of surveys distributed; number returned and analysed; response rate	Distributed: 35 933; analysed: 5054; response rate: 14%	Distributed: not known; analysed: 545 (474 staff, 47 patients, 24 carers); response rate: not known	Survey completed by 929 patients – not known how many Attend Anywhere contacts were made in survey timescale	80 responses (24 in-patient and liaison, 56 community); response rate: 50%
Demographic information for respondents	Not reported	Demographics collected but not reported: gender, age, ethnicity, sexual orientation, disability, religion or belief	Demographics given for all contacts (face-to-face, phone, Attend Anywhere, other) while the survey was live. Age, gender, borough and indices of deprivation, ethnicity, mental health cluster	Not reported

SLaM, South London and Maudsley.

The first theme described the convenience of remote consultations. Some patients appreciated reduced travel, reported less disruption to their work or caring responsibilities, were comfortable at home, and felt they were less likely to miss appointments. However, for other patients, remote consultations were not convenient as they had difficulties with accessing or utilising equipment for video and/or phone calls or accessing a private space, and some people found it more difficult to include their families or carers in consultations. Some staff highlighted the benefit of reduced commuting if working from home, felt it was easier to involve a range of professionals in multidisciplinary meetings compared with face-to-face working, and felt that remote consultations saved time, with benefits for their work–life balance. However, for other staff, remote consultations were not convenient when they did not have the correct equipment, privacy or ergonomic set-up and were described by some as taking more energy, leaving people feeling ‘drained’ and providing less opportunity for breaks or downtime between meetings. Staff noted that remote working reduced informal interactions with team members in which issues could be solved quickly without emails or meetings.

Choice was a key theme. Responses from patients clearly demonstrated that they would like to be given the option of face-to-face or remote consultations. If engaging in a video consultation, patients wanted to choose whether to have their camera on or off. Staff found remote working more acceptable if they felt it was a choice. Staff requested better guidance on when to offer remote or face-to-face consultations.

Different perspectives were expressed on therapeutic alliance. Some patients found it easier to ‘open up’ to a clinician during a remote consultation, although there was a suggestion that some had greater difficulties with this. Likewise, staff reported feeling that some patients were more open during remote consultations and others more withdrawn. Body language is difficult to translate over video and not possible over phone. Some felt they had to compensate for this, which was a burden.

The final theme related to longer-term use of remote consultations. At the time the surveys were conducted (all between April and August 2020), many people felt that remote consultations were a safe way of accessing care in terms of infection control. Many patients expressed that they might be open to remote consultations for some appointments in the future. A number of staff members who were working from home hoped that this could continue for a portion of their working week.

We had very limited information on the demographic characteristics of people who returned surveys (as questions on demographics were often not asked), limiting our ability to comment on whose views are and are not represented. However, the findings of the surveys probably underrepresent the views of those who are least likely and able to engage in remote consultations – this is an important limitation of the analysis. We also recognise that the surveys were designed to capture a snapshot of perspectives at a particular point in time within the context of the acute phase of a pandemic and that viewpoints may shift.

A key output from workstream 2 was the subsequent development of freely downloadable core survey questions that can be tailored according to the needs of the service and used to assess the quantity and quality of NHS appointments undertaken by phone or video or face to face.^12^ It is hoped that this resource may enhance system-wide coordination and impact of research and evaluation efforts by encouraging consistency in approach, leading to establishment of a common minimum data-set that will create a robust understanding across different services.

#### Survey of projects on remote working (workstream 3)

Responses to the electronic survey described the aims and methods of 32 projects intending to investigate remote consultations in mental health services, spanning south London, north-east London and north-central London, UK. The MOMENT group used the survey findings to identify remaining research and evaluation gaps that would not be addressed by the planned projects and to be aware of evidence that may emerge in the future. Responses described 16 service evaluation projects, five quality-improvement projects, five projects with elements of both service evaluation and quality improvement, five research projects and one strategy discussion. The broad methodological approaches used were: survey (20 projects); mixed methods (seven projects); interviews and/or focus groups (three projects); analysis of routinely available data (one project); and unclear (one project). The majority of projects (29 of 32) sought to assess patient and/or staff perspectives on experience and/or access via surveys or interviews. Just over a third of projects (11 of 32) stated the intention to involve patients or members of the public in the project team, for example, in aspects of project planning and delivery, as opposed to involving patients and/or carers as participants in the project. Under a third (nine of 32) signalled an intention to collect demographic information from participants. These points were recognised by the MOMENT group as important gaps in terms of understanding for whom remote working does and does not work well. A small minority of projects (four of 32) set out to assess the effects of remote working on clinical outcomes or examine cost. None of the projects assessed cost-effectiveness. The majority of respondents did not report applying a specific implementation framework, theory or model to guide their planned project.

### Commonalities across workstreams: data synthesis

Thematic analysis of findings across the three workstreams produced the following interrelated themes.

#### Acceptability

Findings from the organisation-wide surveys mirrored findings from the two literature reviews in terms of the acceptability of remote working to patients, carers and staff. Both workstreams suggest that although there are different opinions, and face-to-face contact may be preferred, remote service delivery can be acceptable to patients, carers and staff, at least in the short-term, with many participants indicating that they were satisfied with this way of working. Levels of satisfaction may be higher when video calls are used as opposed to telephone calls. Notably, the pre-COVID umbrella review indicated that these findings may apply outside the context of a pandemic. It is not straightforward to draw conclusions regarding whether particular mental health conditions are more or less suited to remote consultations. One of the patient surveys suggested that the majority of respondents accessing a service for Children and Young People found remote appointments ‘better’ or ‘OK for me’. One of the evaluations within workstream 2 suggested that remote appointments were most likely to be used for patients in the least severely ill clusters. The during-COVID systematic review found that remote consultations may be less feasible and acceptable for some clinical presentations, including some (but certainly not all) patients with psychosis, learning difficulties or autism. However, a tailored offer and personal choice are key. Findings from the staff and patient surveys, in particular, illustrated the point that individuals may find remote consultations acceptable on some occasions or in some circumstances but not others. The likelihood of non-response bias is a key caveat here, as participants across workstreams who were both able and motivated to engage in research and provide their feedback or data may not be representative of wider populations. Reflections on one of the patient surveys, administered by telephone within an older adults service and a learning disability service, suggested that anecdotally those who were less happy with remote consultations were less inclined to complete the survey.

#### Accessibility

For many people, the widespread adoption of remote technologies at the start of the pandemic removed choice and reduced their ability to access mental health services. For example, the staff and patient surveys demonstrated that some patients had received text messages inviting them to a video-based consultation and including a link to join the virtual meeting, without any prior conversation about whether this format was appropriate for their needs. Similarly, survey responses described consultations offered via telephone without assessment of whether this was an appropriate means of communication for individual patients and carers. Some organisations developed decision-making guidance to help clinicians choose whether to offer remote, in-person or blended consultation.

#### Convenience

We define convenience as the ability to engage with remote consultations without difficulty. Three of the systematic reviews included within the pre-COVID umbrella review assessed convenience, with most patients indicating that engaging with therapy sessions from home via remote interventions was convenient. Convenience was also a main theme arising from the thematic analysis of patient and staff survey findings. Many respondents highlighted the convenience and time-saving and/or money-saving nature of remote consultations due to not needing to travel. Further, some people felt that remote appointments could facilitate the attendance of more people from a multidisciplinary team, although others suggested the opposite was true. Importantly, however, there was a consistent message that some people find remote consultations inconvenient some or all of the time. Patients cited difficulties with computer literacy, having an appropriate private space, involving family members or carers in appointments where this was wanted, and poor virtual meeting etiquette (e.g. being left in ‘waiting rooms’ for lengthy periods). Staff also noted problems with meeting etiquette (e.g. meetings over-running), unsuitability of the environment for privacy and ergonomic reasons, and the tiring nature of virtual meetings. One of the staff surveys and the during-COVID systematic review both indicated that assessing new patients by telephone was particularly challenging owing to an inability to see non-verbal cues.

#### Therapeutic alliance

Findings from both the staff and patient surveys and the systematic evidence reviews suggested that for some people it is possible to develop a good therapeutic alliance remotely, although it is perceived that therapeutic alliance may be better when consultations are delivered face-to-face. In the pre-COVID umbrella review, female older adults and US military veterans generally expressed a preference for talking to therapists in person. One of the studies included in the during-COVID systematic review reported that 88% of clinicians found it more difficult to establish a therapeutic relationship with new clients when consultations were held remotely. Similarly, two systematic reviews within the umbrella review included findings demonstrating poorer clinician ratings of therapeutic alliance during remote work. There was some suggestion that therapeutic alliance may develop more easily in consultations held using video-conferencing software as opposed to telephone.

#### Technological challenges

Within the patient and staff survey findings, specific issues relating to the use of technology included: user confidence and knowledge around using technology; issues with wi-fi and connectivity; ability to access (and cost of) appropriate equipment and software subscriptions; and security and/or information governance challenges. Having access to technology and appropriate support to use this technology were identified as key barriers to uptake. These findings applied across patients, carers and staff. Three of the systematic reviews included in the pre-COVID umbrella review mentioned technical difficulties as a challenge, although none of these implied that technical difficulties had been a severe barrier to implementation. However, issues were reported around mistrust in technology, low image resolution and connectivity problems.

#### Exclusion

We did not have systematically collected data to demonstrate the extent of digital exclusion or to draw conclusions about which groups of people are most adversely affected. However, findings across workstreams raise the possibility that many people may have been excluded from accessing mental health services, or have had their access reduced, as a result of the rapid shift to remotely delivered services. This is mirrored by the presumed exclusion of people who do not routinely use remote technologies from much of the research and evaluation data that have been analysed to date. A small number of projects (of which we obtained details via the workstream 3 e-survey) sought to understand the perspectives of some groups who are potentially more likely to be digitally excluded (for example, people with learning disabilities and older adults); it will be informative to see the findings when they are available. Within the staff and patient survey synthesis, it was recognised that the perspectives of older adults are mostly unknown. Although the umbrella review included data relating to some groups thought more likely to be digitally excluded (e.g. older adults), there was a lack of evidence for other groups, including children and young people and in-patients, and overall, as outlined above, there was a lack of demographic information about people who had participated in the research studies (which was a key limitation in the data across the workstreams).

#### Guidelines

Responses to staff surveys indicated that staff would appreciate clear guidelines on how and when to offer remote as opposed to face-to-face consultations. This was echoed in the during-COVID systematic review. The umbrella review included one systematic review of guidelines for video-conferencing-based mental health treatments.^13^ This review encapsulated guidance on decisions about the appropriateness of remotely delivered mental health services, ensuring competence of mental health professionals, legal and regulatory issues, confidentiality, professional boundaries and crisis intervention. One of the collaborating NHS trusts in the MOMENT partnership subsequently developed a decision-making tool to help staff to consider whether remote, face-to-face or a mix of these is the best approach for individual patient consultations.^14^

#### Perspectives are not universal

There are a variety of perspectives regarding remote consultations between staff and patients. The remote delivery of mental health services works well for some people but not others and is appropriate in some situations or on some occasions but not others for many individuals. An individualised approach offering choice is vital. This range of preferences and requirements exists for patients, carers and staff. The effects of remote consultations on a range of outcomes – for example, clinical symptoms for patients, or well-being or productivity for staff – may not be universal either; this too remains to be established.

## Discussion

### Findings in relation to the wider literature

In accordance with our findings, reviews of the literature from mental health settings,^15–17^ physical health secondary care settings^18,19^ and general practice^20,21^ all suggest that remote consultations can be acceptable to some patients and staff but strongly recommend maximising patient choice by offering both remote and face-to-face options. Views are mixed and changeable.^22^ There is a clear call in the literature to ‘keep what works’, as remote consultations can be a convenient and cost-effective way of receiving care for many (but not all).^15,16^

Digital exclusion is a key concern,^23^ with groups most affected by related health inequalities including people of older age,^24,25^ people on low incomes, Black and minority ethnic communities, disabled people, those living in rural or deprived areas, those who face severe and multiple disadvantages (for example homelessness, substance misuse and mental ill health)^25^ and people with severe mental illness.^26^ However, some authors comment that remote consultations have the potential to reduce inequity for some people (lowering time and distance access barriers and tailoring of communication according to language and literacy needs) as well as widening inequalities for others.^16,27^ Resources aimed at improving access to digital mental health services have been developed.^28^

Variation in the confidence of staff members delivering remote consultations is evident.^20,29^ Reviews spanning numerous healthcare settings including mental health demonstrate that staff want more guidance, retrospective training, support and regulation for remote consultations, in particular owing to worries about clinical risk if important mental and physical signs and symptoms are missed.^20,29–31^

### Research and evaluation gaps

#### Reaching those who are least able to engage in remote consultations

All three workstreams are likely to underrepresent the voices of those who are least likely and able to engage remotely. Data collection mechanisms to date have been overly reliant on electronic means – for example, surveys administered by email. Future research must proactively reach digitally excluded people and enable their participation.^23^

#### Uptake

Some of the data considered by the MOMENT group suggest that the offer and uptake of remote consultations varies according to service. Further work could be done to understand the reasons underpinning choices made by services and differences in uptake among different patient groups.

#### Change over time

We were unable to draw conclusions about whether perceptions in relation to remote consultations have changed over time or whether viewpoints will evolve. Longitudinal data are needed to inform future choices and investments.

#### Blended models of service delivery

We currently know little about the experiences and effectiveness of mental health services that are delivered through a combination of remote and face-to-face consultations. Research is needed to evaluate the implementation of new pathways including hybrid or blended approaches to service delivery (a mix of face-to-face and remote delivery) and de-implementation of old ways of working.

#### Effectiveness

Although the pre-COVID-19 umbrella review demonstrated that remotely delivered services could be as good as face-to-face appointments in improving clinical outcomes in some circumstances, we cannot say with certainty whether this finding holds true in the case of fast and widespread implementation owing to the pandemic, as there was a lack of high-quality quantitative evidence within the during-COVID-19 systematic review. It will be important for future work to address questions of clinical effectiveness.

#### Cost and cost-effectiveness

Given the relative dearth of evidence on effectiveness within the pandemic context, little is known about the cost-effectiveness of remotely delivered mental health services. Within the umbrella review, two systematic reviews examining costs or cost-effectiveness met our inclusion criteria. One of these concluded that remote psychiatric interventions could be cost-effective compared with face-to-face interventions, particularly in rural areas where the number of consultations required before remote delivery becomes more cost-effective (outweighing initial equipment costs) is lower.^32^ In the second systematic review which looked at costs, 60% of included studies reported that remotely delivered programmes were less expensive than in-person care, for reasons including savings in travel time and reduced need for patients and their families to take time off work.^33^ However, eight studies in this review concluded that remote programmes were more expensive, particularly owing to video-conferencing equipment costs, and a final study found no difference in costs. Further research regarding costs and cost-effectiveness is needed, particularly as video-conferencing software is now more widely available, and the increase in acceptance of remote consultations precipitated by the pandemic may mean that a greater proportion of patients wish to take up a remote offer (on some occasions) in the future.

#### Implementation effectiveness and support

The COVID-19-specific literature review focused on exploring barriers and facilitators to optimal implementation of remote working, and the emerging evidence on this is summarised. The pandemic led to remote consultations and remote work being implemented urgently as a matter of need and not choice. This presented little chance to study implementation effectiveness in real time; thus, work remains to be done to establish best practices in terms of implementing remote consultations. Such studies are now feasible, as remote options are being offered on an ongoing basis in many settings. The existing implementation science literature may be helpful in designing better implementation support going forward. Furthermore, we may be able to apply frameworks retrospectively to generate additional learning from implementation efforts undertaken within the context of a crisis.

### Strengths and limitations

A significant limitation of the synthesis presented here is the lack of data from those who are digitally excluded. Pandemic restrictions meant that data collection methods were limited and highly reliant on the internet or text messaging. Furthermore, sparse demographic data were available across the workstreams.

Particular strengths of the work include the cross-organisational collaborative approach taken, the inclusion of the perspectives of those who deliver and have received remote mental healthcare, and the inclusion of experts by experience in the project team who have blogged about their experience of working on the project.^34^

### Reflections on the MOMENT partnership approach

The MOMENT group sought to better understand the impact of remotely delivering consultations in mental health services, to inform decisions by healthcare providers on whether to embed or adapt remote delivery going forward. The collaboration between mental health service providers, experts by experience, service and innovation developers and academics was effective in gathering international and local information and synthesising and sharing knowledge about remote consultations locally and nationally to inform ways of working and evidence-based adjustments. The partnership was therefore successful in meeting the four original project objectives. This could be considered the first step in the development of a ‘learning healthcare system’ or ‘learning health network’ which emphasises ‘communication, collaboration and coordination among institutions’.^3^ Next steps could involve continuing to meet to share learning or evaluating how changes to the remote offer are made on an ongoing basis. This would depend on the continuous collection of information and communication of knowledge across the system, requiring sustained participation from collaborating partners.^3,35^

## About the authors

**Lucy Goulding** is at King's Improvement Science, King's College London, London, UK. **Julie Williams** is at the Centre for Implementation Science, King's College London, London, UK. **Alison White** is at Health Innovation Network, London, UK. **Aileen Jackson** is at Health Innovation Network, London, UK. **Zoë Lelliott** is at Health Innovation Network, London, UK. **Stuart Adams** is at South West London and St George's NHS Trust, London, UK. **Kia-Chong Chua** is at the Centre for Implementation Science, King's College London, London, UK; and South London and Maudsley NHS Foundation Trust, London, UK. **Noushig Nahabedian** is at South London and Maudsley NHS Foundation Trust, London, UK. **Juliana Onwumere** is at South London and Maudsley NHS Foundation Trust, London, UK; the NIHR Biomedical Research Centre at South London and Maudsley NHS Foundation Trust and King's College London, London, UK; and the Department of Psychology, Institute of Psychiatry, Psychology and Neuroscience, King's College London, London, UK. **James Woollard** is at Oxleas NHS Foundation Trust, London, UK. **Nick Sevdalis** is at King's Improvement Science, King's College London, London, UK; and the Centre for Implementation Science, King's College London, London, UK. **Fiona Gaughran** is at South London and Maudsley NHS Foundation Trust, London, UK; the NIHR Biomedical Research Centre at South London and Maudsley NHS Foundation Trust and King's College London, London, UK; and the Department of Psychosis Studies, Institute of Psychiatry, Psychology and Neuroscience, King's College London, London, UK.

## Data Availability

The data that support the findings of this study are available upon reasonable request and with permission of F.G.
